# Treatment-free remission in MS: long-term disease control with cladribine tablets

**DOI:** 10.1007/s00415-026-13753-w

**Published:** 2026-03-18

**Authors:** Heinz Wiendl, Ralf Gold, Refik Pul, Michael Ernst, Markus C. Kowarik, Juliane Klehmet, Ines Siglienti, Michael Hübschen, Torsten Wagner, Judith Knaup, Christoph Kleinschnitz

**Affiliations:** 1 Department of Neurology and Neurophysiology, University Medical Center, Freiburg, Germany; 2 Department of Neurology, St. Josef-Hospital Bochum, Ruhr University Bochum, Gudrunstr. 56, 44791 Bochum, Germany; 3 Department of Neurology and Center for Translational Neuro- and Behavioral Sciences, University Medicine Essen, Essen, Germany; 4 Neurology Practice Sinsheim, Sinsheim, Germany; 5 Department of Neurology & Stroke, and Hertie-Institute for Clinical Brain Research, Eberhard-Karls University of Tübingen, Tübingen, Germany; 6 Center for Multiple Sclerosis, Department of Neurology, Jüdisches Krankenhaus Berlin, Berlin, Germany; 7 Merck Healthcare Germany GmbH (an affiliate of Merck KGaA), Weiterstadt, Germany

**Keywords:** Cladribine, Immune reconstitution, Multiple sclerosis, Remission, Treatment-free

## Abstract

Oral cladribine, a highly effective pulsed selective immune reconstitution therapy (SIRT) for relapsing multiple sclerosis (RMS) is characterised by extended treatment-free periods following brief exposure to medication. Since approval in 2017, long-term real-world data have become available which provide insight into the management of patients treated with cladribine tablets beyond year 4. Most patients remained without additional therapy, which may hint at stable disease control. However, the absence of further treatment must not necessarily be interpreted as absence of any disease activity, as MRI data are often incomplete in watch-and-wait cohorts. The observed long-term remission is likely linked to the unique mode of action, which involves rapid repopulation of lymphocytes with different dynamics amongst subsets and sustained reduction of memory B cells. The recovery of the immune system and lymphocytes preserves long-term therapeutic options with existing or upcoming drugs. In cases of mild recurring disease activity, retreatment with cladribine tablets has been shown to be effective and tolerable within the known safety profile. Unrestricted long-term management options associated with cladribine tablets include a switch to other DMTs in cases of insufficient disease control. Overall, cladribine tablets show potential of a paradigmatic shift in MS management that may enable treatment-free remission over 6 years as an achievable treatment goal in a substantial proportion of RMS patients.

## Introduction

**Fig. 1:**
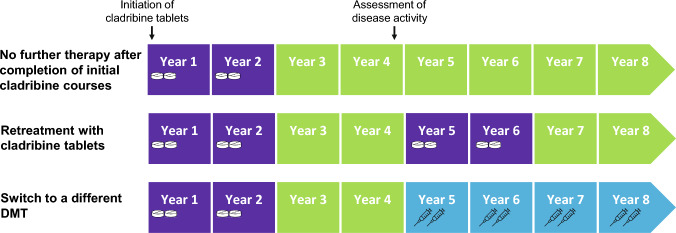
Different therapy management strategies of cladribine-treated patients beyond year 4. Each treatment course of cladribine tablets consists of 2 treatment weeks. Cladribine tablets are administered in two treatment courses approximately one year apart. In the first year, month 1 starts any time followed by month 2 four weeks later of the respective year. Each treatment week consists of 4 or 5 days on which a patient receives 10 mg or 20 mg (one or two tablets) as a single daily dose, depending on body weight. *DMT* disease-modifying therapy

Oral cladribine is a highly effective pulsed selective immune reconstitution therapy (SIRT) licenced for relapsing multiple sclerosis (RMS) in Europe since 2017, followed by other countries. A full treatment cycle covers 4 years and comprises two treatment courses given one year apart, followed by 2 treatment-free years. This concept of pulsed SIRT involving extended periods without drug exposure is characterised by a low burden of treatment and monitoring, which offers patients flexibility in family planning and vaccinations. The mode of action limits the number of lymphocytes transitioning into the CNS via selective depletion of dividing and non-dividing T and B cells [1]. Rapid repopulation of lymphocytes results in recovery of the immune system and maintained immune competence [2], whereby the time to repopulation varies amongst lymphocyte subsets (CD4 + T cells, 43 weeks [3]; regulatory T cells, 34 weeks [4]; B cells, 30 weeks [3]). Long-term control of disease activity has been linked to the sustained reduction and subsequently altered clonal composition of memory B cells [1, 5, 6], which has been observed for up to 7 years [7–10]. Since approval of cladribine tablets, an increasing number of patients are already in their 5th to 9th year following therapy initiation. Consequently, more data on the effectiveness and safety of cladribine tablets are becoming available, along with insights into the management of cladribine-treated patients beyond year 4. At the threshold into unchartered territory, experts had proposed three treatment strategies for year 5: (1) Extending the treatment-free period under structured monitoring, (2) starting a new four-year cycle with cladribine tablets, or (3) switching to another high-efficacy therapy with a different mode of action (Fig. 1). The decision regarding which strategy to pursue was to be based on the patient’s individual profile in terms of presence or absence of disease activity in year 4, disease activity at initiation of cladribine tablets, age, tolerability, and patient preferences [11, 12]. This narrative review discusses existing and emerging long-term data on effectiveness and safety for cladribine tablets in MS up to year 8 after treatment initiation and provides an overview on the feasibility of implementing the three management strategies into clinical practice.

## Methods

This narrative review is based on a comprehensive non-systematic review of the existing literature, which was screened using the terms “cladribine” AND “long-term” AND “real-world”. All studies published between 2020 and 2025 were included. In addition to peer-reviewed studies as the primary sources, other relevant literature was obtained by screening congress abstracts from major meetings of MS expert associations in order to provide recent insights where peer-reviewed data were not yet available.

### Long-term and new real-world data on cladribine tablets’ efficacy and safety profile

After conclusion of the CLARITY Extension trial (NCT0064153), which provided evidence of long-term disease control without further treatment courses in years 3 and 4 [13], patients were further observed under controlled conditions in the clinical study CLASSIC-MS, the Italian MS registry CLARINET-MS, the prospective open-label phase IV trials MAGNIFY-MS, CLARIFY-MS, and the prospective observational long-term safety registry PREMIERE. Assessment of efficacy parameters such as annualised relapse rate (ARR), expanded disability status scale (EDSS), magnetic resonance imaging (MRI), progression independent of relapses (PIRA), cognition, quality of life (QoL), and patient satisfaction indicated a durable effect beyond year 4, with a substantial proportion of patients (58.1%) requiring no further treatment with disease-modifying therapies (DMTs) [14]. No new safety signals have emerged [15].

Since marketing authorisation in 2017, a total of 131,017 patients, amounting to 367,021 patient-years, have been treated with cladribine tablets by June 2025 [16]. Adverse events (AEs) from post-approval sources (including spontaneous individual case safety reports [ICSRs], noninterventional post-marketing studies, and data from clinical trials and other solicited sources) continuously collected in the pharmacovigilance database are annually published in periodic safety update reports (PSURs). Table 1 provides a summary of reported adverse events of special interest. Data for long-term risk after retreatment are limited at this point in time. Assessing the subgroup of older patients receiving retreatment separately would be valuable. Neither an elevated long-term malignancy risk, secondary autoimmunity, nor infection risk has been observed so far. Real-world long-term data up to 8 years since the first patients initiated cladribine tablets are meanwhile available. Table 2 provides an overview of current cladribine cohorts and their key findings. In terms of baseline characteristics, these cohorts are heterogeneous, but generally match those of an expected RMS population, where women are about thrice more often affected than men and patients are usually diagnosed before the age of 40 years. The proportion of female patients ranges from 58% [17] to 87% [18]. The reported mean age ranges from 36 [19] to 52 years [14] and the median EDSS score ranges from 2.0 [19–21] to 3.5 [14]. Between 15% [21] and 80% [15] of patients received cladribine tablets as first-line DMT, and most pre-treated patients had received one or two prior DMTs [22, 23].

**Table 1:** Cumulative adverse events of special interest (as of 07 July 2025) [16]

AEs of special interest	Adjusted reporting rate^a^ per 100 patient-years, (95% CI)
Serious infections (1549 reports)	0.42 (0.40; 0.44)
Herpes zoster (1002 reports)	0.27 (0.26; 0.29)
Malignancies^b^ (518 reports)	0.14 (0.13; 0.15)
Liver injury (736 reports)	0.20 (0.19; 0.22)
Serious lymphopenia (437 reports)	0.12 (0.11; 0.13)
Opportunistic infections (excluding PML^c^ and tuberculosis) (46 reports)	0.01 (0.01; 0.02)
Tuberculosis (42 reports)	0.01 (0.01; 0.02)
Seizures (199 reports)	0.05 (0.05; 0.06)

*AE* adverse event, *CI* confidence interval, *PML* progressive multifocal leukoencephalopathy

^a^The reporting rate is adjusted for the cumulative duration of patient exposure to cladribine tablets

^b^The spectrum of malignancies resembled the distribution of cancer types seen in the general population, without any clustering of specific tumour types

^c^As of 07 July 2025, there were no confirmed cases of PML related to oral cladribine

**Table 2:** Overview of Long-term data

Study	Follow-up, design	N^a^	Key findings	
			Effectiveness	Safety
CLASSIC-MS [14]	10.9 years (median), ambispective, multicentre	160	58.1% (93/160) no further DMT after initial CT courses; sustained (median time since last CT dose: 10.9 years) long-term mobility and disability benefits: 88% EDSS < 7; 79% EDSS < 6	Not assessed
CLARINET-MS [17]	11.4 years, retrospective, registry-based	80	At month 60, 57.2% relapse-free; according to KM estimates, 63.7% free of disability progression; 67.5% switch to another DMT; estimated probability of remaining treatment-free at month 60 was 28.1%	Not assessed
MAGNIFY-MS Extension [22]^c^	4 years, prospective, multicentre	219	79.20% (95% CI 72.30, 84.56) achieved NEDA-3 during year 4; 83.1% (n = 172) and 67.4% (n = 126) had no T1 Gd + lesions and no active T2 lesions in year 4, respectively; ARR of 0.09 over 4 years; stable or improved SDMT in year 4 achieved by 79.0% (4-point change) and 88.1% (8-point change)	3 patients (1.4%) had ≥ 1 serious TRAE: infections (3 events), neoplasms (1 event)
CLARIFY-MS Extension [23]^c^	4 years, prospective, multicentre	280	68.6% had no or minimal decline^b^ in cognitive function (SDMT) from baseline to Month 48; stable or improved SDMT in year 4 achieved by 63.9% (4-point change) and 77.5% (8-point change), low ARR over 4 years (0.13); stable number of T1 Gd + lesions over 4 years; mean (± SD) cumulative number of new or enlarging T2 lesions in year 3 + 4: 2.1 (± 4.55)	TRAE documented in 15 patients; TRAE affecting > 1 patient: urinary tract infection (n = 3), herpes zoster (n = 2), malignancies (n = 2)
PREMIERE [15]	8.9 years, prospective, registry-based	923	Not assessed	10 cases of malignancies; serious infections and infestations documented in 23 patients
Real-world data - Germany				
IQVIA, claims data [28]^c^	6 years, retrospective, prescription-based	1350	853 (63.2%) no further DMT after initial CT courses; 278 (20.6%) retreatment with CT; 219 (16.2%) switch	Not assessed
Kleinschnitz et al. [21]	6 years, retrospective, multicentre	166	105 (63.3%) no further DMT after initial CT courses; 50 (30.1%) retreatment with CT in year 5; 11 (6.6%) retreatment with CT before year 5, 90% were relapse-free in year 5 and EDSS was stable in 73% and improved in 3%	After 3rd CT course: No grade 4 lymphopenia, no bilirubin or ASAT elevations, grade 1 ALAT elevations (n = 3), infections (n = 6), no malignancies related to CT
Kowarik et al. [26]	5 years, retrospective, multicentre	187	118 (63.1%) no further DMT after initial CT courses; 36 (19.3) retreatment with CT in year 5; 35 (18.7%) switch	Not assessed
Ernst et al. [24]^c^	8 years, retrospective, multicentre	125	The majority of patients remained free of disease activity without additional treatment after initial CT courses	No severe lymphopenia or herpes zoster infections after additional CT courses in years 5 + 6
NIS CLIP-5 [31]^c^	5.5 years, prospective, multicentre	70	The study investigated retreatment with CT in year 5; the decision for retreatment with CT in Year 5 was made in most cases (70%) by default to extend disease control or prevent disease reactivation, not due to acute clinical or paraclinical symptoms	No grade 4 lymphopenia after CT retreatment
German MS registry [25]^c^	7 years, registry-based	282	52.5% Watch & Wait, 9.9% retreatment, 28.0% switch within 4 years, 9.6% switch after year 4; ARR activity: decrease during years 5 and 6 in “watch&wait” and “restart” groups, but increase in year 7; decrease in year 7 amongst „switch-out “; MRI activity: Increase from years 5 to 7 in “watch&wait”, decrease over time in “restart” and “switch-out”	Not assessed
Konen et al. [33]	720 days (median) after switch, retrospective, multicentre	42	The study investigated highly active RMS patients who switched from CT to anti-CD20 antibodies; patients benefitted from the switch, which led to effective disease stabilisation (reduction in relapse activity from 83 to 25%, reduction in MRI activity from 58 to 10%, increase in proportion of achieving NEDA-3 from 5 to 53%)	Mean lymphocyte counts remained within the normal range
Real-world data - International				
NIS CLADRISE [29]^c^, Czechia	4 years, prospective, registry-based	255	60.8% no additional treatment, 92.3% of those were relapse-free; 19.6% retreatment with CT; 19.6% switch to another DMT	Not assessed
Oreja–Guevera et al. [32]^c^, Spain	5–7 years, retrospective, single centre	59	60% have received three courses of CT; 17% four courses, 40% remained untreated beyond Year 2; 15% switched to another DMT	Additional CT courses were well tolerated, with 25% reporting mild adverse events, mainly fatigue and headache (35%). No cases of grade 4 lymphopenia, opportunistic infections, or significant liver toxicity were observed
Maroto-Navas et al. [18]^c^, Spain	5 years, retrospective, single centre	86	93.3% relapse-free, 90% no MRI lesions in Year 4;	Side effects were mild and transient, with mild infections (52.56%) being the most frequently reported, and no cases of grade 4 lymphopenia were observed
PMS IMSE10 [37]^c^, Sweden	≥ 3 years, registry-based	194	Clinical stability and significant improvements in ARR and T2 lesions; 93% did not switch to another DMT	Not reported
CLADCOMS [38]^c^, Sweden	3 years, prospective, multicentre	147	78.2% relapse-free; 3% new T2 lesions; clinical stability after 36 months regardless of previous treatment	Not reported
Rauma et al. [19], Finland	4 years, retrospective, registry-based	191	Estimated treatment persistence at 4 years was 70%; mean ARR 0.2	No grade IV lymphopenia and only one case of herpes zoster reactivation (0.5%) were reported
Budimkić et al. [20]^c^, Serbia	5 years, prospective, single centre	306	Treatment naive patients had better chance for treatment response (odds ratio 2.68 (CI 0.88–8.15), NEDA-3 achieved by 89%, 80.3% and 74% in Years 2–4, respectively	Not reported
Alferes et al. [27]^c^, Portugal	5–7 years, single centre	45	57.6% treatment-free, 39.4% retreatment, 3.0% treatment-switch	The lymphocyte profile following CT retreatment was similar to the first and second course (mild to moderate lymphopenia) and no serious adverse events occurred
MSBase registry [30]^c^, Australia	6 years, registry-based	3834	Treatment-naïve PwMS had a significantly longer time to first relapse (HR 0.72; 95% CI 0.58, 0.89, p = 0.003). 4.6%, 4.8%, 10.3%, and 4.9% were retreated with CT in Years 3–6	Not reported
Arun et al. [39], UK	5 years, single centre	3	The case series investigated retreatment with CT in year 5; no evidence of disease activity at 6 months after retreatment	No new infections or any other AE related to CT

*ARR* annualised relapse rate, *CI* confidence interval, *CT* cladribine tablets, *IMSE10* immunomodulation and multiple sclerosis epidemiology 10, *KM* Kaplan–Meier, *MRI* magnetic resonance imaging, *NEDA-3* no evidence of disease activity (no relapses, no progression independent of relapse activity, no isolated MRI activity), *PMS* post-marketing surveillance, *SD* standard deviation, *SDMT* symbol digit modalities test

^a^Patient overlap between some studies within the same country cannot be ruled out. ^b^Defined as an improved or stable SDMT score or a decline of ≤ 4 points in the SDMT score, at 4 years after initial dose of CT (Month 48) compared to SDMT score prior to initial dose of CT

^c^Validity may be limited due to extraction from congress abstracts

#### Watch-and-wait approach

**Fig. 2:**
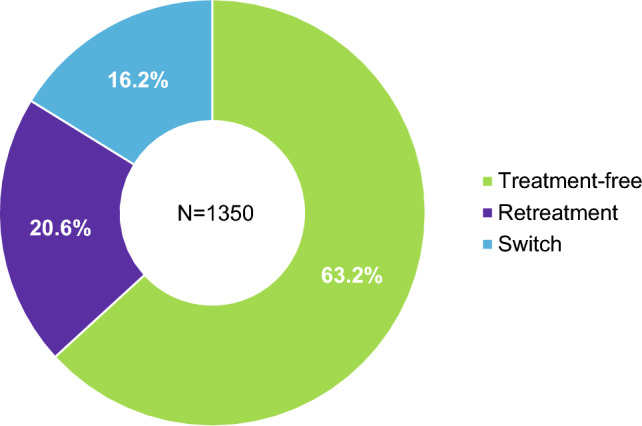
Treatment status in year 6 following initiation of cladribine tablets, based on data from the IQVIA longitudinal prescription database of prescription information in Germany [28]

Data from German [21, 24–26] and Portuguese [27] real-world cohorts and the German prescription database (IQVIA) [28] have shown that the watch-and-wait approach is the most frequently applied strategy (52.5%–67.8%), probably due to the prospect of maintained control of disease activity following the initial two courses of cladribine tablets (Fig. 2). High proportions of patients experiencing freedom from disease activity in terms of clinical symptoms (relapse activity, EDSS progression, Multiple Sclerosis Functional Composite) and MRI findings in year 5 support this approach [18, 20, 21, 24, 29] (Table 2). In this context, a better response to cladribine tablets in terms of time to relapse was observed in treatment-naive patients compared to those who received prior DMTs [20, 30]. Using Cox proportional hazard regression to compare clinical outcomes between treatment-naive and pre-treated groups, treatment-naive patients had a significantly longer time to first relapse (HR 0.72; 95% CI 0.58, 0.89, *p* = 0.003), whilst no difference was found between the groups in 24-week confirmed disability progression (HR 0.92; 95% CI 0.67, 1.27) or 24-week confirmed disability improvement (HR 1.13; 95% CI 0.79, 1.61) [30].

#### Retreatment with cladribine tablets

A smaller proportion (19.3%–39.4%) were retreated with cladribine tablets in years 5 and 6 [21, 26–29]. The ongoing observational study CLIP-5 investigated these patients with RMS who received the full dosage of cladribine tablets according to the summary of product characteristics (SmPC) in years 1 and 2 and were retreated with cladribine tablets in years 5 and 6. Retreatment occurred in most cases (70%) by default to extend disease control or prevent disease reactivation. Symptomatic disease reactivation, clinically evaluated by EDSS and relapse rate, was the reason for retreatment in 15.7%, whilst 14.3% experienced non-symptomatic disease reactivation as evaluated by MRI. Retreatment was effective: By month 18 after retreatment, 59 of 70 (84.3%) patients were relapse-free. Median EDSS remained stable at 2.0 over the 5.5 years following initiation of cladribine tablets [31]. Tolerability after retreatment was consistent with the safety profile known from the initial treatment courses. Lymphopenia, observed in 21 out of 71 patients, occurred in line with the mechanism of action, yet no grade 4 lymphopenia (< 200 × 10^9^ cells/L) after retreatment with cladribine tablets has been reported. As second most frequent related treatment-emergent adverse events, nausea, headache and alopecia were each reported in two patients [31]. In concurrence with data from CLIP-5, retreatment with cladribine tablets in other real-world cohorts did not trigger any serious adverse events attributable to the therapy, including grade 4 lymphopenia [21, 24, 32].

#### Switch to another DMT

Switching to another high-efficacy therapy is another option in case of recurring disease activity and has been performed by 3%–19.6% of patients under real-world conditions [27–29, 32]. A retrospective data analysis focussing on patients switching from cladribine tablets to anti-CD20 antibody therapies showed that the switch led to effective disease stabilisation [33]. A disproportionally high number of patients switching to another DMT was found in the CLARINET-MS study (67.5%). These switches were mostly protocol-mandated as the study recruited patients from previous RCTs (CLARITY, CLARITY Extension, ONWARD, and ORACLE-MS) [17]. Patients who converted from clinically isolated syndrome to clinically definite MS in ORACLE-MS had to switch to subcutaneous interferon beta according to the study protocol [34, 35]. In the ONWARD trial, patients received cladribine in addition to interferon beta, and a protocol-mandated switch was triggered by occurrence of lymphopenia [36].

### How do we know which strategy to pursue?

#### Patients with stable disease control

Patient-related baseline factors such as age and disease activity at initiation of cladribine tablets need to be taken into account in the decision-making process beyond year 4 [12].

The long-term data reviewed above support durable effectiveness of cladribine tablets beyond year 5. The high proportion of patients receiving no further treatment due to effective disease control after completion of the initial two cladribine courses indicates that an extended period of treatment-free remission may be a feasible goal in a substantial number of RMS patients. Particularly older patients (age > 50 years) have been shown to achieve effective long-term relapse control, which supports the use of cladribine tablets as a viable treatment exit strategy in this population [40]. Thus, in case of absence of clinical and paraclinical disease activity by the end of year 4, continuing the treatment-free period may be considered in patients at low risk of disease reactivation. Close monitoring to detect returning relapse and MRI activity is advisable in these patients [25].

#### Patients at risk of disease reactivation

Younger patients and/or patients with high activity at baseline tend to experience disease reactivation earlier. Accordingly, the watch-and-wait approach is not advisable in these cases. However, retreatment with cladribine tablets appears to be an effective option to maintain long-term disease control in patients at risk of disease reactivation. Retreatment indications in real-world studies included radiological activity, mild clinical activity, unspecific symptoms, poor prognostic factors, such as high disease activity at baseline [32] or increased sNfL [27]. In addition, patient wish and retreatment by default to extend disease control or prevent disease reactivation have been mentioned as reasons for retreatment [31]. No cases of grade 4 lymphopenia, opportunistic infections, or significant liver toxicity were observed and tolerability was in line with the known safety profile established in the clinical trials [32]. While this strategy has been endorsed by experts within the German disease-oriented competence network multiple sclerosis (KKNMS) [41], opinions differ in other countries. In Finland, for instance, retreatment with cladribine tablets is not recommended due to concerns of herpes zoster reactivation [19]. However, the risk of herpes zoster can be mitigated by proactive measures that include vaccination, treatment administration at adequately recovered lymphocyte counts, as well as close monitoring during the acute treatment period [42].

#### Patients with insufficient disease control

Switching to another DMT with a different mode of action is a feasible option for the subset of patients who do not achieve sufficient disease control or experience disease reactivation by the end of year 4 or earlier. Indications for switching DMTs include persistent or new inflammatory disease activity either clinically or on brain or spinal cord magnetic resonance imaging (MRI). In the real-world studies listed in Table 2, the proportion of patients with disease reactivation in year 4 ranged from 3% [38] to 26% [20]. The time to disease reactivation varies between patients. Possible patterns and a potentially predictive role of biomarkers remain topics of future research. The recovery of the immune system and lymphocytes preserves long-term therapeutic options with existing or upcoming drugs. Switching active RMS patients from cladribine tablets to anti-CD20 antibody therapies has been demonstrated to be safe and effective, indicating complementary mechanisms of actions [33].

## Outlook and conclusion

Treatment with cladribine tablets offers a unique concept (SIRT) that has been associated with prolonged periods of disease control without additional treatment in a subset of RMS patients. This potential of treatment-free remission over 6 years as an achievable treatment goal could lead to a paradigmatic shift in MS management. However, findings on sustained efficacy beyond year 4 should be interpreted in light of the selected study populations as observational cohorts are generally subject to selection bias. Survivor bias, another limitation inherent to long-term data, is potentially minimised by reporting the proportion of patients who switched to another therapy. In this context, absence of further treatment must not necessarily be interpreted as absence of any disease activity, considering that MRI data are often incomplete in most watch-and-wait cohorts. Nevertheless, a potential association cannot be ruled out, as it is clinically plausible that the presence of disease activity would prompt either retreatment with cladribine tablets or a switch to another DMT. Thus, absence of further treatment may imply disease stabilisation. The term ‘treatment-free remission’ describes the absence of measurable disease activity in this context. There is a need for a unified definition of its assessment which should be developed as consensus amongst experts in the field. Retreatment with cladribine tablets in years 5 and 6 has primarily been driven by ongoing disease activity or unfavourable prognostic markers, including high baseline disease activity, lesion burden, and elevated NfL or GFAP levels, underscoring that treatment-free remission is not universally achieved. Notably, data from the German MS registry show an increase in ARR in year 7 in both the watch-and-wait and retreatment groups [25]. This finding underlines the necessity of close monitoring. Treatment with cladribine tablets allows for flexible long-term management, including the option to switch to alternative DMTs, which is relevant given interindividual variability in treatment response [33]. The safety profile is well documented in clinical trials, with adverse events occurring exclusively in context of cladribine tablets administration and being generally transient [24]. Accordingly, an extended period of treatment-free remission may be anticipated to correspond to a period free of adverse events. Nevertheless, long-term safety conclusions remain limited by the duration of available follow-up, and the assumption that treatment-free periods are entirely free of adverse events warrants cautious interpretation. A closer look at the subgroup of older patients receiving retreatment could provide valuable insights into the long-term malignancy risk, secondary autoimmunity, and infection risk in this vulnerable group. The recovery and repopulation of lymphocytes is a key characteristic of cladribine, linked to its mode of action. Some patients, however, experience persisting lymphopenia (< 1% grade 3; ~ 12% grade 2 in year 4), which warrants regular monitoring [21]. Of note, some of the data presented here are limited by small patient numbers and extraction from congress abstracts. Confirmation in peer-reviewed journals is eagerly awaited within the next few years. The majority of the real-world studies presented here are registry-based or multicentre studies; thus investigator bias is minimised. Due to the lack of controlled clinical trials with unified inclusion criteria, this review relies on real-world cohorts. While these cohorts are heterogeneous in terms of baseline disease activity, prior treatments, and follow-up intensity, the pattern of proportions of patients remaining treatment-free, undergoing retreatment or switching is comparable. As the cohorts mature, further analyses will be conducted to explore the potential of treatment-free remission in the subset of patients at low risk of reactivation and the long-term safety of patients receiving retreatment. With time and more patients reaching years 5–8, a stratification by baseline characteristics may lead to the identification of prognostic characteristics. Additional biomarkers could potentially contribute to prognostic assessment, but their role remains exploratory. In conclusion, consistent across clinical trial extensions and real-world cohorts totalling a large number of patients (> 6000), a major proportion (52.5%–67.8%) achieved sustained disease control up to 8 years, so that no further treatment was necessary following the initial doses of cladribine tablets.

## Data Availability

No new data were generated or analysed in the course of this review. All data referenced are available in the cited sources.
